# Total Biomass in the Neotropical Savanna Domain: Stock Estimation and Modeling with Edaphic Variables

**DOI:** 10.3390/plants15152261

**Published:** 2026-07-24

**Authors:** Kennedy Nunes Oliveira, Eder Pereira Miguel, Alba Valéria Rezende, Eraldo Aparecido Trondoli Matricardi, Aldicir Osni Scariot, Ricardo de Oliveira Gaspar, Matheus Santos Martins, Evelyn Bianca Almeida Vaz, Diego Martins Stangerlin, Leonardo Job Biali, Álvaro Nogueira de Souza

**Affiliations:** 1 Department of Forest Sciences, Faculty of Technology, University of Brasília (UnB), Brasilia 70297-400, DF, Brazil; edermiguel@unb.br (E.P.M.); alba.rezende@gmail.com (A.V.R.); ematricardi@unb.br (E.A.T.M.); aldicir.scariot@embrapa.br (A.O.S.); ricogaspar@unb.br (R.d.O.G.); eflevelyn@gmail.com (E.B.A.V.); diego.stangerlin@unb.br (D.M.S.); ljbiali@unb.br (L.J.B.); ansouza@unb.br (Á.N.d.S.); 2 Ecology and Conservation Laboratory, Embrapa Genetic Resources and Biotechnology, Brasilia 70770-901, DF, Brazil; 3 National Forest Inventory, Brazilian Forest Service (SFB), Brasilia 70818-900, DF, Brazil; matheus.martins.28@gmail.com

**Keywords:** belowground biomass, Cerrado, litter, necromass, roots, trees, tropical soils

## Abstract

In the Neotropical Savanna domain, few equations are available for estimating biomass stocks in Cerrado *sensu stricto* (CSS), despite the importance of such tools for estimating carbon stocks, understanding ecosystem functioning, and supporting conservation actions. We conducted forest inventories in 40 temporary 1000 m^2^ plots in southeastern Brazil to estimate total and compartmental biomass stocks and to model biomass using structural and edaphic predictors. Total biomass (TB) included aboveground woody biomass (AGWB), necromass, litter, and belowground biomass (BGB). AGWB was estimated for trees with basal diameter ≥ 5 cm using a previously fitted regional equation. Root biomass was sampled using a 1 m^3^ trench excavated at a single point adjacent to each plot. Necromass was quantified using the line-intersect method along a 50 m transect, considering debris with diameter ≥ 3 cm. Litter was sampled using a 0.25 m^2^ frame placed at the center of each plot. Biomass was modeled on an area basis using a hierarchical approach for TB, total tree biomass (TTB = AGWB + BGB), and AGWB. Mean stocks (Mg ha^−1^ ± s.d.) were 45.24 ± 17.32 (TB), 20.47 ± 11.26 (AGWB), 18.47 ± 10.88 (BGB), 5.49 ± 4.18 (litter), and 0.81 ± 1.62 (necromass). Models selected using the Akaike Information Criterion (AIC) and validated by repeated k-fold cross-validation achieved rŷy = 0.76, 0.72, and 0.94 and RMSE = 24.40%, 27.06%, and 18.10% for TB, TTB, and AGWB. As the equations were calibrated for CSS under the environmental conditions of the Brazilian semiarid region, their transferability to other Cerrado regions should be considered with caution. The inclusion of soil variables (e.g., Al, Mg, and sand) improved predictions, reducing relative costs and taxonomic dependence, while incorporating nutritional adaptations to the acidic soils characteristic of the biome.

## 1. Introduction

Recent estimates indicate that more than 40% of all tree species on Earth occur in South America [1], highlighting the central role of the continent in global biomass and carbon stock dynamics. This Neotropical region also harbors important savanna formations, such as the Cerrado, the Brazilian savanna [2]. At the global scale, savannas represent an important terrestrial carbon reservoir, particularly if protected from fire and grazing, which would increase their potential as carbon sinks [3]. However, these ecosystems have historically received less attention in the context of ecosystem services [4], and most biomass estimates remain strongly based on forest formations. In this context, studies quantifying total biomass in Cerrado vegetation formations are still scarce [5], with most research restricted to woody biomass [6], which limits an integrated understanding of biomass stocks and their incorporation into ecological models.

The Brazilian Neotropical savanna is the second largest biome in South America and is characterized by a mosaic of vegetation formations with distinct structural and floristic compositions [7], strongly influenced by fire regimes [8]. It is also among the most tree-diverse regions in the world [9]. The Cerrado has been recognized for at least two decades as a global biodiversity hotspot [10,11], whose structural diversity results in high environmental heterogeneity [7,12].

This heterogeneity is expressed in marked environmental gradients along the distribution of the biome, distinguishing different biogeographic regions. In studies of biomass modeling at the plot scale in the Cerrado, variation among regions has influenced biomass stocks and the performance of predictive models [13]. In particular, Minas Gerais includes a portion of the Cerrado inserted within the Brazilian semiarid region [14], whose environmental conditions differ from those prevailing in the Cerrado of Central Brazil, especially in terms of water availability and climatic seasonality [15]. These differences may influence vegetation structure and biomass allocation and, consequently, the performance of biomass estimation models developed in other regions of the biome.

In this context, studies conducted in these vegetation formations present important methodological challenges, particularly for estimating total biomass stocks, including the high frequency of multi-stemmed individuals that complicates the application of standard diameter-based allometric approaches, the scarcity of locally developed allometric equations, and the high uncertainty associated with belowground biomass estimates.

One of the most comprehensive models for estimating aboveground tree biomass in tropical vegetation is the pantropical model proposed by Chave et al. [16], which includes diameter at breast height, tree height, and wood density. However, wood density for woody species in the Cerrado is an underdocumented variable [17,18], which limits the applicability of this equation in many situations. Therefore, developing biomass equations specific to Cerrado conditions is essential.

In this regard, alternatives that reduce dependence on taxonomic information, such as wood density, become particularly relevant. The development of simple and specific models that incorporate soil variables into the modeling process is advantageous, as it integrates edaphic variables that reflect the nutritional adaptations of native vegetation to the acidic soils characteristic of this biome [19]. This approach is also advantageous because it reduces the relative cost compared to obtaining wood density, requires only a minimal set of soil analyses (rather than full edaphic assessments), and overcomes logistical limitations associated with sampling, especially belowground. Furthermore, recent studies have highlighted the existence of a coupling relationship between soil and vegetation, indicating that both components influence one another over time [20]. In this context, edaphic variables are mechanistically associated with vegetation structure, individual abundance, and, consequently, biomass stocks [21].

Although pantropical equations often show good statistical fit, this apparent accuracy may mask an important limitation which is the aggregation of multiple biomes into a single dataset. The literature indicates that this lack of local specificity may generate biases when applied to data outside the original sample and also highlights the underrepresentation of large trees in calibration datasets [22].

Furthermore, because differences among sites are averaged out at the global scale, a pantropical allometric equation may exhibit local bias even when it is globally valid. The fact that the residual errors of these equations are not substantially larger and may even be smaller than those of local models suggests that pantropical equations do not always adequately capture allometric particularities associated with local environmental conditions [23]. Therefore, whenever possible, the use of equations developed for local conditions is recommended [24].

Given this, the choice of model structure is also an important aspect in the biomass modeling process. Simple predictive models tend to be preferable because they are easier to test in replication and validation trials, require fewer data to estimate parameters reliably, are less sensitive to influential statistics and collinearity, and allow for a more biologically meaningful interpretation of their parameters [25].

Regarding modeling approaches, most studies focus on aboveground biomass and are conducted at the individual tree level, relying heavily on dendrometric variables such as tree height, whose field estimation is often associated with considerable uncertainty, in addition to the substantial architectural variation among species. Furthermore, these models generally disregard the influence of edaphic factors on biomass variation. However, can the inclusion of soil variables improve estimates of total biomass, total tree biomass, and aboveground woody biomass per unit area in the Neotropical Savanna domain when compared with models based solely on basal area?

As an alternative, the present study aimed to fit and evaluate biomass models per unit area for total biomass (TB), total tree biomass (TTB), and aboveground woody biomass (AGWB) in Cerrado *sensu stricto* (CSS) areas, incorporating basal area and soil physical and chemical variables in the northern and northwestern regions of the state of Minas Gerais, Brazil.

## 2. Results

### 2.1. Biomass Stocks and Soil–Vegetation Relationships

The average productivity (Mg ha^−1^ ± s.d.) in the CSS areas was 45.24 ± 17.32 for total biomass, 20.47 ± 11.26 for woody biomass, 5.49 ± 4.18 for litter, 0.81 ± 1.62 for necromass, and 18.47 ± 10.88 for root biomass. For root biomass, the maximum root diameter observed using the trench method was 5 cm.

These stocks corresponded to a tree density of 920 individuals ha^−1^ and a basal area of 8.07 ± 4.07 m^2^ ha^−1^, with an absolute sampling error of 1.30 m^2^ ha^−1^ and a relative sampling error of 16.12%, excluding dead individuals, in dystrophic soils (V < 50%) with approximately 70% mean sand content (Table 1).

The high variability observed is associated with the strong structural and environmental heterogeneity of the studied CSS. For litter, this variation may reflect seasonal deposition processes, resulting in highly irregular spatial distribution and point-based sampling within a single plot. For necromass, the high variability is related to episodic mortality events and woody debris input, as well as the occurrence of plots with no necromass records, which contributes to zero values and increases heterogeneity among sampling units. For belowground biomass, the high variability is associated with the well-known difficulty of sampling this compartment, given its subterranean nature.

The families with the highest number of individuals in the community were Vochysiaceae (201 ind. ha^−1^), Fabaceae (182 ind. ha^−1^), and Myrtaceae (152 ind. ha^−1^). In terms of species richness, Fabaceae was the richest family (32 species), followed by Vochysiaceae (11 species) and Myrtaceae (9 species).

Among the 150 species recorded in the community, only the ten species with the highest biomass stocks accounted for 62.9% of the woody biomass. Among these ten species, eight are tolerant to aluminum in acidic soils, including five aluminum-accumulating species (*Eugenia dysenterica*, *Qualea parviflora*, *Qualea grandiflora*, *Callisthene fasciculata*, and *Vochysia thyrsoidea*), three aluminum-excluding species (*Caryocar brasiliense*, *Hymenaea stigonocarpa*, and *Tachigali subvelutina*), and two species for which no solid information on aluminum tolerance was available (*Terminalia fagifolia* and *Machaerium opacum*).

These aluminum-tolerant species represented 36% of the total importance value (IV%) (27.3% and 8.7% for accumulators and excluders, respectively) and 52.6% of the woody biomass (31.3% and 21.3% for accumulators and excluders, respectively) (Table 2).

### 2.2. Biomass Modeling per Unit Area

For total biomass (TB) modeling, three models were fitted: two selected using the stepwise procedure, with the lowest Akaike Information Criterion (AIC), and one simple model based solely on basal area (G) (Table 3). For total tree biomass (TTB) modeling, only two models were retained, since the second-best model obtained through the stepwise procedure was identical to the simple model based on G.

For aboveground woody biomass (AGWB), although model selection was also conducted using MLR (Multiple Linear Regression), the final models were fitted using non-linear regression due to evidence of non-linear relationships between predictor and response variables and funnel-shaped residual patterns.

For the TB estimation models, when comparing the $\Delta_{i}$ of the best model (M1) with that of the simple model (M3), which uses only G as a predictor variable, a difference greater than 10 (16.36) was observed. Thus, the simple model was considered to have essentially no empirical support [31,32], indicating that it was not competitive and that model M1, which includes soil variables, was indeed the best option given the dataset. Both models (M2 and M3) presented $\Delta_{i}$ > 2 (Table 4).

Model M1 improved TB estimates, reducing RMSE% to 24.40% and increasing the correlation between predicted and observed values (rŷy = 0.76), compared to 31.47% and 0.55 for model M3, for RMSE% and rŷy, respectively.

The intermediate model M2 presented $\Delta_{i}$ greater than 4 (7.10) and was classified as having considerably less empirical support relative to the best model [31]. Its RMSE% was 27.34%, and the correlation between predicted and observed values was rŷy = 0.69.

**Figure 1:**
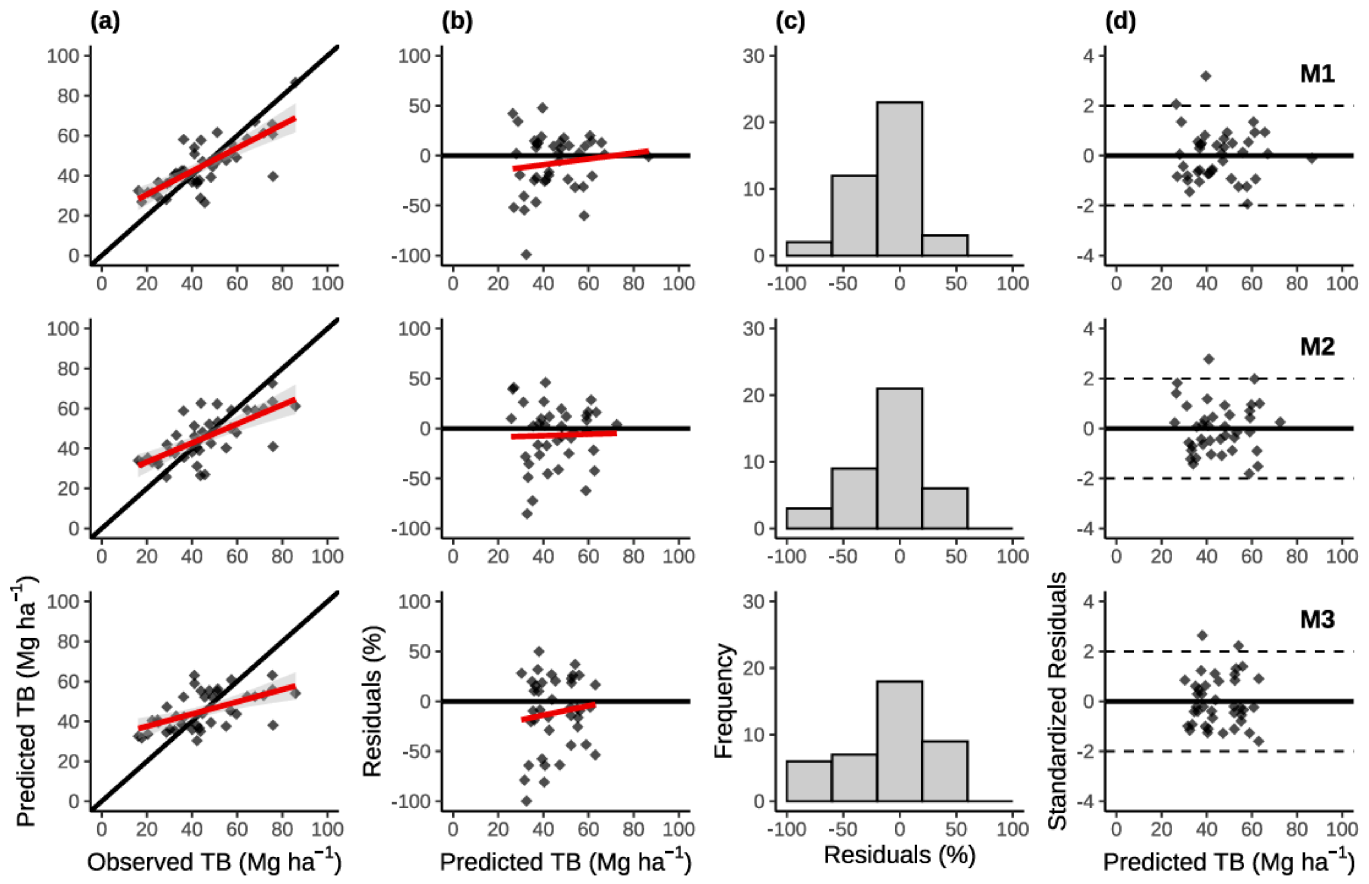
Observed versus predicted values (**a**), residuals versus predicted values (**b**), histogram of residual classes (**c**), and standardized residuals versus predicted values (**d**) for total biomass (TB) models M1–M3 (Table 4), per unit area, in Cerrado *sensu stricto* (CSS) in the northern and northwestern regions of Minas Gerais, Brazil. The red line represents the trend line.

The graphical analysis of residuals (Figure 1) showed that all three models tend to over-estimate total biomass stocks. In addition to the variable G, the multiple linear regression performed using the stepwise method identified magnesium (Mg) and aluminum (Al) as the soil variables that most influenced the estimation of total biomass. The bias associated with the back-transformation of aboveground woody biomass estimates was corrected by applying the correction factor proposed by Sprugel. Total biomass was calculated as the sum of the biomass compartment estimates, and its uncertainty was obtained through error propagation considering the variances and covariances among compartments.

**Figure 2:**
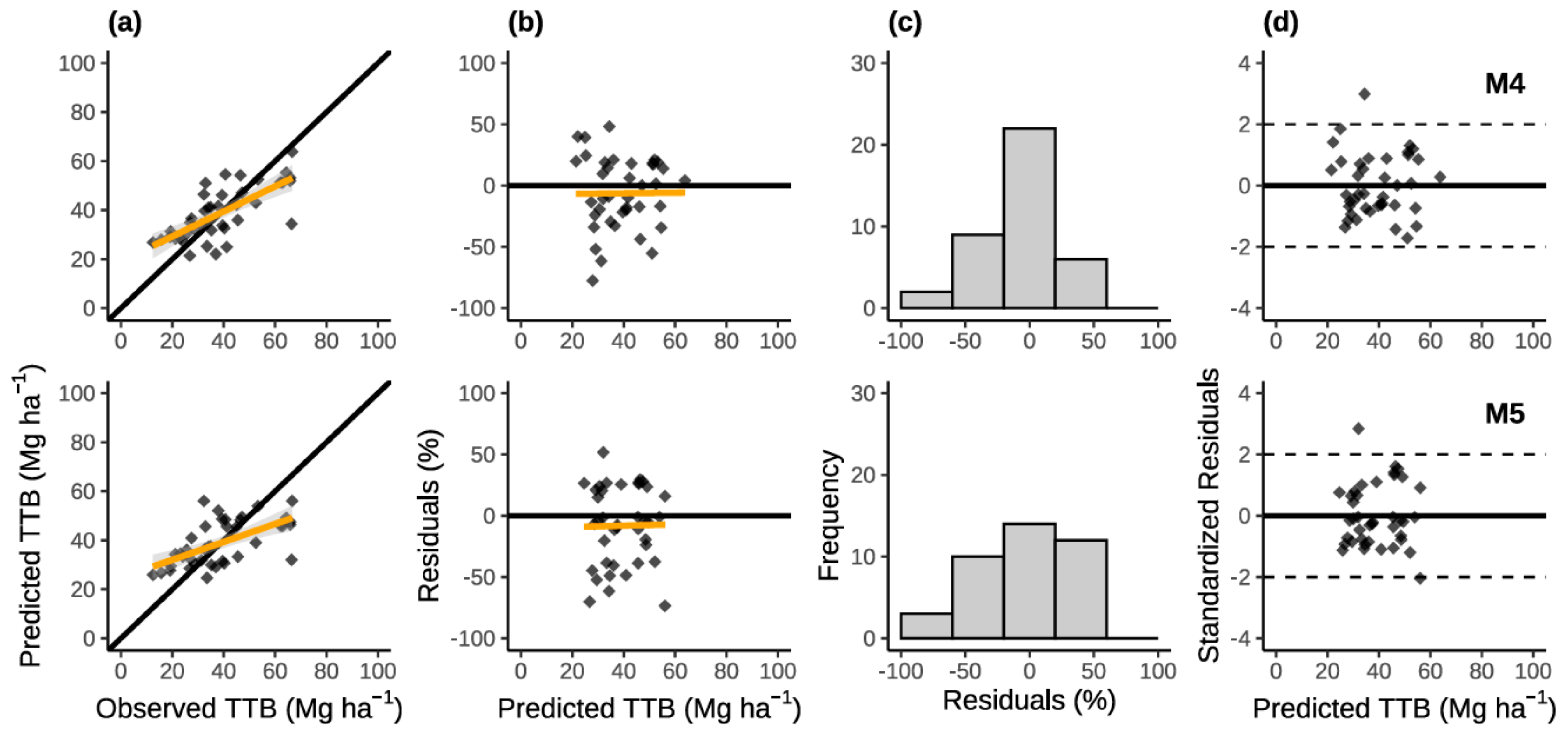
Observed versus predicted values (**a**), residuals versus predicted values (**b**), histogram of residual classes (**c**), and standardized residuals versus predicted values (**d**) for total tree biomass (TTB) models M4 and M5 (Table 4), per unit area, in Cerrado *sensu stricto* (CSS) in the northern and northwestern regions of Minas Gerais, Brazil. The yellow line represents the trend line.

For TTB modeling, the second-best model selected by the stepwise method corresponded to the simple model; therefore, only two models were evaluated (M4 and M5) (Figure 2). The $\Delta_{i}$ difference between the best model (M4) and the simple model (M5) was well above 2 ($\Delta_{i}$ = 8.49), indicating that the simple model is not competitive and has essentially low empirical support, considering the dataset used (Table 4).

Once again, the estimates were improved, with a reduction in RMSE% from 30.85% (M5) to 27.06% (M4) and an increase in the Pearson correlation between predicted and observed values (rŷy) from 0.61 to 0.72 for models M5 and M4, respectively. In addition to the variable G, the multiple linear regression conducted using the stepwise method identified magnesium (Mg) as the main additional predictor in the estimation of total tree biomass.

For AGWB modeling, the $\Delta_{i}$ difference between the best model (M6), which presented the lowest AIC, and the simple model (M8) was 16.79, thus greater than 10, indicating a difference not attributable solely to the inclusion of additional variables. The simple model (M8) was considered to have essentially no empirical support.

**Figure 3:**
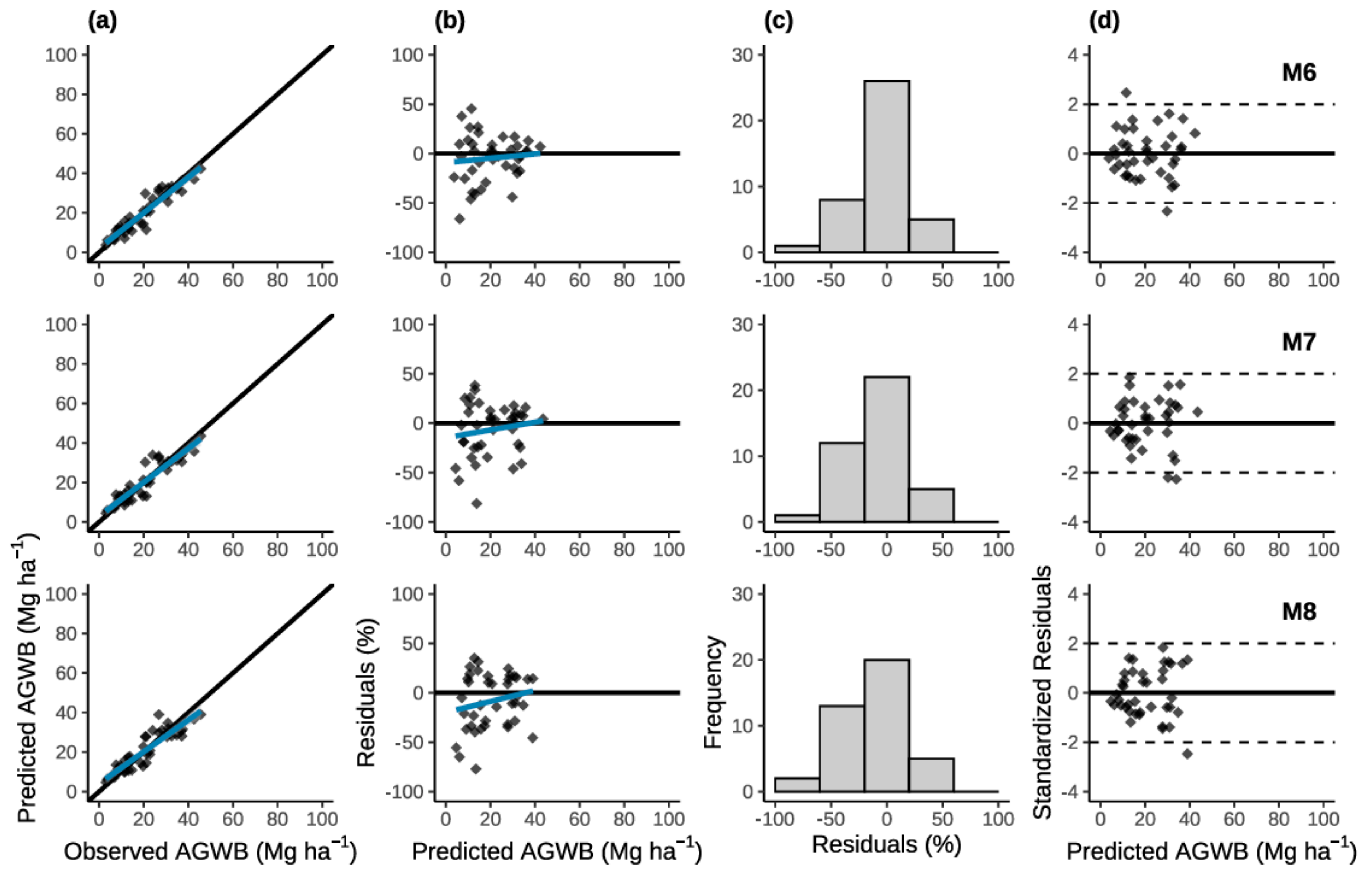
Observed versus predicted values (**a**), residuals versus predicted values (**b**), histogram of residual classes (**c**), and standardized residuals versus predicted values (**d**) for aboveground woody biomass (AGWB) models M6–M8 (Table 4), fitted per unit area, in Cerrado *sensu stricto* (CSS) in the northern and northwestern regions of Minas Gerais, Brazil. The blue line represents the trend line.

Estimates were improved, with a reduction in RMSE% to 18.21% and a correlation between predicted and observed values (rŷy) of 0.94 for model M6, compared to 23.48% and 0.90 for model M8, respectively (Table 4). Accuracy in terms of predicted and observed values was similar, all above 90%, despite a slight improvement with the inclusion of soil variables (Figure 3).

The intermediate model (M7) presented $\Delta_{i}$ greater than 2 (7.69). According to Burnham and Anderson [31], this indicates that model M6 has substantial empirical support and differs from model M7 not only due to the presence of an additional parameter (sand). If the AIC difference were less than 2, the model with fewer parameters should be selected [32,33], which was not the case. The performance of model M7 was also only slightly superior to that of the simple model, with a small reduction in relative error.

In addition to the variable G, the multiple linear regression conducted using the stepwise method identified magnesium (Mg) and sand as the main soil variables associated with the estimation of woody biomass for the studied Cerrado areas.

Thus, for estimating TB stocks, the best model was M1. For TTB stocks, the best selected model was M4. And for AGWB stocks, the most suitable model was M6. These models showed correlations between predicted and observed values of 76%, 72%, and 94%, and relative errors (RMSE%) of 24.40%, 27.06%, and 18.10%, respectively.

In this study, all variance inflation factors (VIFs) were close to 1 for total biomass and total tree biomass modeling, whereas for woody biomass they were close to 5. However, these values are still within acceptable practical limits (<10) [34,35], indicating no evidence that collinearity was a problem (Table 4). All selected biomass estimation models showed significant *F*-values (*p* < 0.01).

Regarding model validation, RMSE analysis showed that, for all models and modeling levels, the RMSE obtained by k-fold cross-validation was higher than the fitted RMSE, resulting in a positive bias (Table S1). This behavior was expected, as cross-validation estimates the prediction error using data not included in model fitting. The observed versus cross-validated predicted value plots were constructed using the mean out-of-fold predictions obtained across the 100 cross-validation repetitions, providing an assessment of the models’ predictive performance on independent data (Figures S1–S3).

The fitted equations obtained in the present study are presented below (Table 5).

## 3. Discussion

### 3.1. Biomass Stocks

In Central Brazil, the sum of above- and belowground fractions, sampled down to 2 m depth, ranged from 21.8 Mg ha^−1^ in “campo sujo” to 77.8 Mg ha^−1^ in “cerrado denso” [36]. The values obtained in the present study (approximately 45 Mg ha^−1^) (Table 1) fall within this range and correspond to a basal area of 8.07 m^2^ ha^−1^ and a density of 920 individuals ha^−1^, excluding dead individuals. However, it is important to note that the study used for comparison included root sampling down to 2 m depth, whereas in the present study roots were sampled only down to 1 m, which may result in an underestimation of belowground biomass relative to the values reported in the literature. It is worth noting, however, that few studies provide joint estimates of above- and belowground biomass fractions, which limits direct comparisons among studies.

The structural diversity of vegetation types in the Neotropical Savanna results in wide variation in biomass stocks. In addition, methods for biomass estimation are diverse, and few studies have quantified these stocks considering different compartments [37].

In Minas Gerais, in areas of CSS, Ribeiro et al. [6] reported biomass stocks of 62.96 Mg ha^−1^ and 37.51 Mg ha^−1^ for trees and roots, respectively, values higher than those observed in this study. This difference is associated with higher tree density (2086 individuals ha^−1^), greater basal area (14.90 m^2^ ha^−1^), and methodological differences in the estimates, including the use of different allometric equations, the inclusion of other biomass compartments (such as leaves), and different criteria for inclusion and root classification, as well as differences in tree measurements, which considered diameter at breast height (DBH), unlike the present study, which used diameter measured at 30 cm above the ground (Db). This issue still lacks standardization, as tree architecture and, consequently, the position at which diameter is measured influence model fitting and application [38].

For biomass stock comparisons, methodological discrepancies persist, especially in the quantification of aboveground tree biomass, which is the most frequently reported component in studies [39]. These difficulties range from the standardization of diameter inclusion criteria, whether at breast height (DBH, 1.3 m) or at the base (Db, 0.3 m), with less common variations such as those adopted by Abdala et al. [40] and Ruggiero et al. [41], who used Db ≥ 2 and 3 cm, respectively, to the scarcity of equations, since total biomass estimation is a costly and time-consuming process.

A similar situation occurs for roots [18], which present clear methodological limitations, including the definition of the destructive method to be applied, as well as for necromass, particularly regarding the diameter threshold used to distinguish this component from litter [42].

### 3.2. Biomass Equation

Available biomass equations for CSS are still predominantly developed at the individual tree level, highlighting the persistence of allometric approaches centered on individual plants. At this scale of analysis, models applied to these vegetation formations face important methodological challenges in estimating biomass stocks, particularly due to the high frequency of multi-stemmed individuals, dependence on taxonomic information, the scarcity of local equations, and the need to account for different biomass compartments, especially belowground biomass. Furthermore, the absence of edaphic variables in most models suggests a limited ability to capture environmental variation in biomass, which may constrain the understanding of the ecological factors influencing its distribution across these vegetation formations.

The results indicate that the inclusion of edaphic variables represents an alternative to reduce costs compared to approaches that rely on wood density determination, a larger set of dendrometric variables, and more complex sampling methodologies, including root sampling for belowground biomass estimation, while also reducing taxonomic dependence and broadening the applicability of models in ecological studies [43,44]. This study proposed an alternative approach to estimate biomass per unit area by incorporating soil variables, in contrast to the most commonly used equations, which are restricted to the individual tree level and, in the case of the most comprehensive equation, depend on wood density.

In this context, Ribeiro et al. [6] tested five standard models combining diameter at breast height (DBH), height (H), and wood density (WD), concluding that the model based on DBH and wood density showed the best performance. Consequently, studies estimating biomass in the most common physiognomy of the Neotropical Savanna, CSS [7], often rely on generic wood density databases or data obtained from vegetation types outside the savanna. However, the literature on wood density for Neotropical Savanna species is still limited [17,18].

Within this framework, tree diameter remains the main predictor in models for estimating volume, biomass, and carbon stocks due to its strong correlation with these attributes [12,18]. In the present study, diameter was not used directly as a predictor variable but was incorporated into the calculation of basal area (G), which was included as an explanatory variable and was present in all models.

In addition to diameter, pantropical models require height, which, although improving biomass models, is a variable that is difficult to obtain and is associated with high measurement errors [45]. The model proposed by Chave et al. [16], although widely used, employs DBH as an explanatory variable, differing from recommendations for the Neotropical Savanna, which indicate the use of diameter measured at 30 cm above the ground in savanna areas due to tree architecture [46], an approach adopted in the present study. Morais et al. [38], when fitting models based on 868 destructively sampled trees in CSS and “campo cerrado” areas in Minas Gerais, concluded that the height at which diameter is measured (0.3 or 1.3 m) influenced the fitting of volume and biomass equations, as trees are naturally thicker near the base.

In the absence of robust local models for the biome, generic or pantropical models may still be useful [13], although continuous efforts are being made to fill these gaps and improve local biomass estimates.

Regarding the equations fitted in the present study (Table 5), the results can be considered satisfactory given the high heterogeneity of the dataset, characterized by high species richness and a mosaic of vegetation formations. At the total biomass level, which integrates multiple biomass compartments and therefore exhibits greater variability, model performance was comparable to that reported in studies conducted at the individual tree level in similar savanna environments. These findings are consistent with the high structural and morphological variability inherent to the woody species of CSS [7,13].

The comparison of model performance with previous studies is limited by the fact that most equations developed for the Cerrado have been fitted at the individual tree level, whereas studies aimed at estimating total biomass per unit area remain relatively scarce. In addition, differences in the scale of analysis, the biomass compartments considered, and the environmental characteristics of the evaluated sites reduce the comparability of the results.

The best-performing models included basal area and edaphic variables and yielded root mean square errors (RMSE) ranging from 18% to 30%, as well as correlations between observed and predicted values (rŷy) greater than 0.72. These values fall within the range reported in the literature and, in some cases, indicate performance comparable to that observed in previous studies. For example, Rezende et al. [12], when developing individual tree-level models for volume, biomass, and carbon in the Cerrado of the Federal District, reported standard errors of approximately 30%, highlighting the uncertainty inherent to allometric estimates in savanna ecosystems. In one of the few plot-level approaches developed for CSS, Ribeiro et al. [6] obtained a coefficient of determination of 0.934 using basal area as a predictor of aboveground biomass, emphasizing the potential of this variable as an integrative measure of vegetation structure. The authors also highlighted the usefulness of this approach in situations where stand-level attributes, such as basal area per hectare, are more readily available than detailed information at the individual tree level.

Similarly, Roitman et al. [13] demonstrated that the incorporation of environmental variables can improve the accuracy of biomass estimates across different spatial scales in the Cerrado. The authors highlighted the significant effect of ecoregions on biomass stocks, showing that part of the variability observed across the biome is associated with environmental differences among regions and reinforcing the relevance of these factors for biomass modeling. In a broader context, Chave et al. [16] and Burt et al. [22] emphasized that uncertainties and potential biases remain important challenges for biomass estimates derived from pantropical models, underscoring the need for methodological improvements and for the incorporation of information that better represents local vegetation and environmental characteristics. In this regard, the performance observed in the present study falls within the expected range for tropical savanna ecosystems and highlights the explanatory gains achieved by integrating dendrometric and edaphic variables.

Although this study does not include an economic analysis, it is evident that the use of equations based on diameter and soil variables is advantageous, as it eliminates the need for species identification, especially in tropical forests with high species richness and wide variation in wood density [47]. In addition, soil sampling and analysis are operationally simpler than direct root sampling, regardless of the methodology used, making inventories faster and less costly when compared to obtaining wood density and excavating trenches.

### 3.3. Soil–Vegetation Relationships

**Figure 4:**
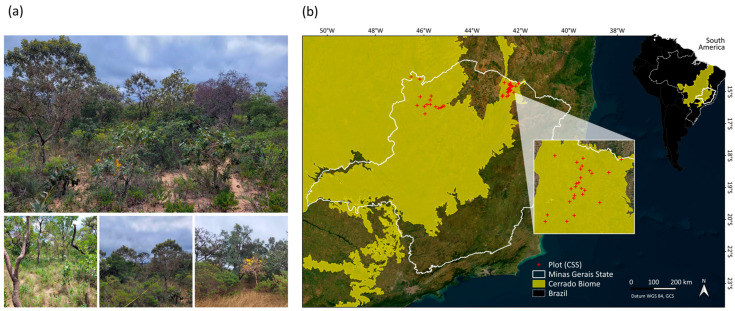
Overview of the landscapes (**a**) and distribution of forest inventory sample plots (**b**) conducted in Cerrado *sensu stricto* (CSS) areas, located in the Pardo and Urucuia River watersheds in the northern and northwestern regions of the state of Minas Gerais, Brazil.

In addition to basal area (G), obtained from the forest inventory, the best-performing models included only the following edaphic variables: aluminum (Al, cmolc dm^−3^), magnesium (Mg, cmolc dm^−3^), and sand content (Sand, %). Regarding magnesium levels, plots with higher biomass stocks were associated with lower concentrations of this nutrient. The studied areas also exhibited high sand content, a characteristic observed visually in the field (Figure 4a) and confirmed by the high average sand content obtained from soil analyses (Table 1). In dystrophic Cerrado soils, low Mg levels may reflect correlated edaphic properties, such as high aluminum saturation, low base saturation, and sandy texture, rather than necessarily indicating a direct effect of magnesium on biomass accumulation [19].

Based on the coefficients of the fitted equations, this study showed a positive correlation between biomass and aluminum (Table 4). At a regional scale, Lopes [48] and Goodland and Ferri [49] reported a negative relationship between biomass and aluminum saturation levels. However, at a local scale, Ribeiro et al. [50], as cited by Ribeiro and Walter [7], observed a positive correlation, consistent with the present study, highlighting the nutritional adaptations of native Cerrado species to acidic soil conditions.

The interpretation of this association requires caution, as it does not necessarily imply a direct beneficial effect of aluminum on plant growth, particularly because the mechanisms responsible for aluminum toxicity are still not fully understood [19]. In highly weathered and dystrophic Cerrado soils, aluminum is often associated with other edaphic attributes, such as low fertility, high acidity, and reduced base saturation. Thus, the positive correlation observed in this study does not necessarily indicate a causal effect of aluminum on biomass accumulation.

Brunner and Sperisen [51] highlight that the high biodiversity and substantial biomass production observed in tropical forests suggest that many woody species are capable of persisting under conditions of high Al^3+^ availability. Following Ryan and Delhaize [52], the term aluminum resistance refers to the ability of plants to grow with little or no injury under elevated concentrations of this element. In the Cerrado, such tolerance has been documented in several woody species. Haridasan [28] demonstrated that various native species accumulate high foliar concentrations of aluminum without exhibiting apparent toxicity symptoms. Subsequently, Haridasan and Araújo [30] showed that this element can be compartmentalized in specific tissues, reducing its potential toxic effects and contributing to the maintenance of physiological homeostasis. In hyperaccumulator species of the Vochysiaceae family, Andrade et al. [53] demonstrated that aluminum can be stored even in chloroplasts without apparent damage to cellular structures. In addition, Bittencourt et al. [54] observed that aluminum exposure induces root exudation of organic acids in Cerrado accumulator species, a mechanism capable of complexing Al in the rhizosphere and reducing its potential toxicity.

In the present study, when considering a subset of the ten most important species, a common practice due to their contribution to most of the biomass, it was observed that Al-accumulating species showed higher phytosociological importance than Al-excluding species. This study follows the definition proposed by Haridasan [28], according to which Al-accumulating plants are those with foliar concentrations exceeding 1000 mg kg^−1^. Although the available classification in the literature is not yet fully complete, with data on tolerance to this element lacking for *T. fagifolia* and *M. opacum* (Table 2), this pattern is consistent with the occurrence of adaptive mechanisms associated with aluminum tolerance in dominant Cerrado species.

Several common Cerrado species, rather than excluding aluminum, absorb and transport it to leaves and seeds. Some even exhibit reduced growth in the absence of this element in nutrient solutions, as observed for *Vochysia thyrsoidea* Mart. (Vochysiaceae) [30], which was among the ten species with the highest woody biomass stocks in the studied areas (Table 2). Regarding the families identified in this study, those with the highest species richness are considered hyperdominant within the biome, with Vochysiaceae, Fabaceae, and Myrtaceae standing out [9]. The genus *Qualea*, which includes *Q. parviflora* and *Q. grandiflora*, both abundant in the study area, is recognized as the most dominant genus in the Neotropical Cerrado [9]. These species have a wide distribution across the biome [55] and are characterized as aluminum accumulators [19,28,29]. The high representation of these taxa in the studied areas is consistent with the occurrence of Al-accumulating species among those that most contribute to the structure and biomass stocks of the evaluated communities.

The reported behavior reinforces the contribution of edaphic variables to the establishment and distribution of vegetation types and, consequently, to biomass stocks in the biome. In addition to the intrinsic physiological adaptations of species, ecological mechanisms mediated by interactions with soil microorganisms may also contribute to plant persistence in chemically restrictive environments. Yu et al. [56] demonstrated that plant–microbe associations can reduce the toxic effects of mercury and promote plant growth under chemical stress conditions. Similarly, He et al. [57] showed that mechanisms related to nutritional homeostasis and antioxidant system regulation enabled the maintenance of seedling growth under cadmium stress. Although these studies were conducted with elements other than aluminum and outside the Cerrado context, they nevertheless indicate that different physiological and ecological mechanisms can contribute to plant tolerance under chemically restrictive conditions.

### 3.4. Study Limitations and Future Research

This paragraph and the following ones address the limitations of the present study, which, like any other, are inherent. For the results obtained, there are inventory errors associated with sampling, which were expressed through the forest inventory analysis considering basal area. There are also errors related to the equation used to estimate woody biomass, which were considered high.

However, it is important to highlight the lack of specific equations for the studied region, as well as the need to rely on species-level wood density databases, given the high floristic richness observed, in order to apply equations developed for adjacent regions or pantropical contexts. In addition, obtaining authorization from environmental agencies for destructive tree sampling represents a political and operational challenge.

Furthermore, there is error associated with modeling per unit area, as shown in the model fitting tables (Table 4). Regarding biomass compartments, root sampling using trenches at a single point per plot may also introduce bias. Nevertheless, the inherent difficulty of sampling the belowground portion of vegetation is widely recognized [58]. The range of root diameters observed was clearly specified and is currently undergoing a transition in terms of the criteria that define fine or coarse roots [59], and the limitations are not only methodological but also financial.

In addition, litter and necromass stocks are strongly influenced by seasonality, showing substantial variation between dry and rainy periods. This aspect is directly related to the temporal stability of biomass stocks and soil properties, since CSS is a highly dynamic ecosystem strongly influenced by recurrent disturbances, especially fire, as well as land-use and land-cover changes, which can significantly alter both biomass and soil attributes over time [60]. Therefore, the fitted equations represent conditions specific to the sampling period and context, and may present limitations when extrapolated to different times of the year or to areas with distinct disturbance histories. These considerations highlight the complexity involved in estimating and modeling biomass, especially roots and, in particular, total biomass stocks.

The potential spatial autocorrelation of soil variables should also be considered, as it may violate the assumption of independence among plots and, consequently, affect the statistical robustness of the inferences. In ecosystems such as CSS, this spatial dependence may reflect natural patterns of edaphic and topographic variation, creating spatial structures that are not fully captured by conventional modeling approaches.

Although the proposed models have been developed for plot-level biomass estimation, their application to regional-scale biomass mapping would depend on the availability of spatially continuous soil information, which remains a challenge in many regions. Future advances in digital soil mapping may facilitate the broader application of this type of model.

Finally, although repeated k-fold cross-validation provides a robust estimate of the predictive performance of the models, external validation remains the preferred approach for assessing their generalization ability. Whenever possible, future studies should validate the models using independent datasets from other regions and/or different seasonal periods, allowing their robustness and stability to be evaluated under a wider range of environmental and temporal conditions.

## 4. Materials and Methods

### 4.1. Study Area

The study was conducted in Cerrado *sensu stricto* (CSS) areas (Figure 4a) [7], within the Pardo and Urucuia River watersheds, located in the northern and northwestern regions of the state of Minas Gerais, Brazil, respectively (Figure 4b).

The Brazilian Rural Environmental Registry System (SICAR) was used to identify the number and geographic location of rural properties within the two watershed areas. These properties are distributed across the municipalities of Vargem Grande do Rio Pardo, Montezuma, Indaiabira, Rio Pardo de Minas, and São João do Paraíso in the northern region of Minas Gerais, and Pintópolis, Urucuia, Riachinho, Bonfinópolis de Minas, Uruana de Minas, Arinos, and Formoso in the northwestern region of the state.

The region is characterized by a tropical hot and humid savanna climate (Aw), with a dry winter season and mean temperature of the coldest month ≥ 18 °C, according to the Köppen–Geiger classification [61,62]. Climatological normals from the National Institute of Meteorology stations for the period 1991 to 2020 indicate a mean annual precipitation of approximately 1100 mm and a mean annual temperature of 25 °C for the northwestern region based on the Arinos station, and 820 mm and 24 °C for the northern region based on the Salinas station [63].

Soils under CSS vegetation are predominantly classified as Oxisols, specifically Red Latosols and Red-Yellow Latosols, according to the Brazilian Soil Classification System [64]. Despite their favorable physical properties, these soils are generally strongly to moderately acidic, with pH ranging from 4.5 to 5.5, and show a widespread deficiency of essential nutrients, particularly phosphorus and nitrogen. They frequently exhibit high aluminum concentrations, and organic matter content ranges from medium to low [7].

### 4.2. Data Collection

#### 4.2.1. Forest Inventory

Temporary sample plots (Figure 4b) were randomly allocated within the study area. Stratification considered the confirmation of the CSS physiognomy, the exclusion of transition zones, and the establishment of only one sampling unit per property in order to reduce spatial autocorrelation among plots. Socio-environmental studies and assessments of silvopastoral systems (SPS) in the study area preceded the allocation of the temporary sample plots [65], such that site selection was mainly conducted in small rural properties that manage cattle within native vegetation, although at low intensity.

**Figure 5:**
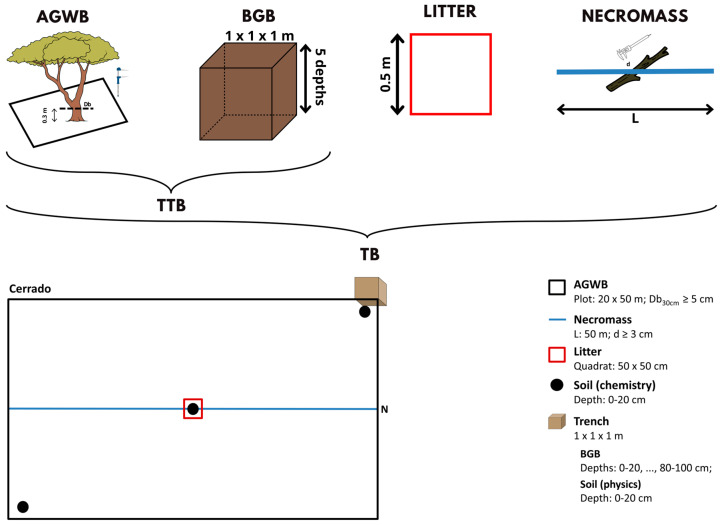
General sampling scheme applied in Cerrado *sensu stricto* (CSS) areas. TB = total biomass; BGB = belowground biomass; AGWB = aboveground woody biomass; TTB = total tree biomass; Db = basal diameter; d = diameter; L = line length.

A total of 40 sample plots were established in CSS areas. Plots of 1000 m^2^ (20 × 50 m) were used. Plot size (Figure 5) was defined according to the Manual of Permanent Plots for the Cerrado and Pantanal Biomes [46]. Field inventories were conducted in March 2024 and 2025 in the northern and northwestern regions, respectively.

All woody individuals with basal diameter (Db), measured at 30 cm above the ground, equal to or greater than 5 cm were recorded and botanically identified according to APG IV [66]. Basal diameter was measured using a diameter caliper. In the case of multi-stemmed individuals, all stems with Db ≥ 5 cm were included in the inventory.

Absolute and relative sampling errors were obtained through statistical analysis of the forest inventory, performed for the basal area variable, considering simple random sampling, according to Péllico Netto and Brena [67].

#### 4.2.2. Estimation of Total Biomass Stock (TB)

To estimate total biomass stock (TB) in each sample plot, both aboveground and belowground (BGB) biomass components were considered. Aboveground biomass included the following components: aboveground woody biomass (AGWB), necromass, and litter, while BGB comprised root biomass.

The uncertainty associated with total biomass was propagated considering the variance of each biomass pool and the covariance among pools, following the general error propagation equation described by Bevington and Robinson [68] (Equation (1)). Thus, the variance of total biomass was estimated as:

(1) $$Var\left(TB\right)=\sum_{i=1}^{n}Var(X_{i})+2\sum_{i<j}Cov(X_{i},\;X_{j})$$

where TB = AGWB + BGB + Litter + Necromass and X represents the biomass pools included in total biomass estimation, with the term $Cov(X_{i},\;X_{j})$ quantifying the degree of non-independence [69].

##### Aboveground Woody Biomass (AGWB)

The aboveground biomass of each woody individual in each vegetation formation was estimated using Equation (2) [38], with back-transformation to the original scale applying Sprugel’s correction factor [70]. The equation considers only stem biomass including bark, determined up to a minimum top diameter of 3.0 cm.

(2) $$AGWB=e^{-10.87846+2.84969\cdot \ln\left(Db\right)}\cdot 1.0085$$

where AGWB = aboveground woody biomass of individual *i* (Mg); Db = basal diameter of individual *i*, measured at 30 cm above the ground (cm); R^2^adj (adjusted coefficient of determination) = 0.94; SEE (standard error of the estimate) = 0.1301 (Mg) and 61.74%.

The dataset used by Morais et al. [38] was derived from the Forest Inventory of Minas Gerais for CSS [71]. In total, 868 trees belonging to 99 species were destructively sampled, distributed across 70 genera and 37 families, in 20 forest fragments located in different regions of the state. Individual tree volume was obtained using the Huber method, and biomass was estimated as the product of volume and basic wood density, determined from disks collected at five positions along the commercial stem height.

##### Necromass

Necromass was estimated using the line-intersect method [72]. For this purpose, a sampling line was established at the center of each CSS sample plot. The line length (L) was set to 50 m.

Due to the numerous terminologies related to deadwood [73], necromass was defined here as woody debris, including branches, stumps, and dead trees or shrubs lying on the ground, that is, non-living woody biomass not accounted for as litter [74]. Thus, all woody debris with diameter ≥ 3 cm intersecting the line (L) or its vertical projection was included in the inventory. The diameter of the debris was measured using a digital caliper. In addition, debris was classified according to the degree of decomposition as (1) fresh, (2) initial decomposition, and (3) advanced decomposition [75,76] (Table 6).

The mass of deadwood for each sample plot was obtained using Equation (3) [72]. Necromass was calculated as the product of volume and the mean basic density (*ρ*) (Table 7) of woody debris intersecting the line (L) in each sample plot:

(3) $$Necromass=\pi^{2}\cdot \frac{\sum_{i=1}^{n}d_{i}^{2}}{8\cdot L}\cdot \rho_{i}$$

where Necromass = deadwood mass (Mg ha^−1^); d_i_ = diameter (cm) of each piece intersecting L; L = line length (m); *ρ*_i_ = mean basic density of woody debris per plot (g cm^−3^), considering diameter and their respective decomposition classes; π/8 = geometric factor derived from probability theory, allowing the intercepted cross-sectional areas to be summed as circles in the line-intersect method, corresponding to the product between π/2, related to the probability of intersection of randomly oriented woody pieces, and π/4, the factor used to convert $d^{2}$ into circular cross-sectional area. This equation embodies three assumptions: that the pieces are randomly oriented, circular in cross section, and horizontal [72].

##### Litter

At the center of each sample plot established for the CSS inventory, a 50 cm × 50 cm frame was installed for litter collection, that is, fine non-woody debris (diameter < 3 cm). Litter from each frame was collected and weighed to obtain total fresh mass. A subsample of the litter from each frame was then taken, weighed, and oven-dried with air circulation and renewal at 70 ± 2 °C until constant mass was achieved (variation ≤ 1%), in order to determine dry mass and thus total dry litter biomass per unit area [78].

Dry litter mass was obtained using the proportionality method [79], according to Equation (4). After determining dry mass, values were extrapolated to megagrams per hectare.

(4) $$DM=WM\cdot \frac{Dm}{Wm}$$

where DM = dry mass of the field sample (g); WM = fresh mass of the field sample (g); Dm = dry mass of the subsample (g); Wm = fresh mass of the subsample (g).

##### Belowground Biomass (BGB)

**Figure 6:**
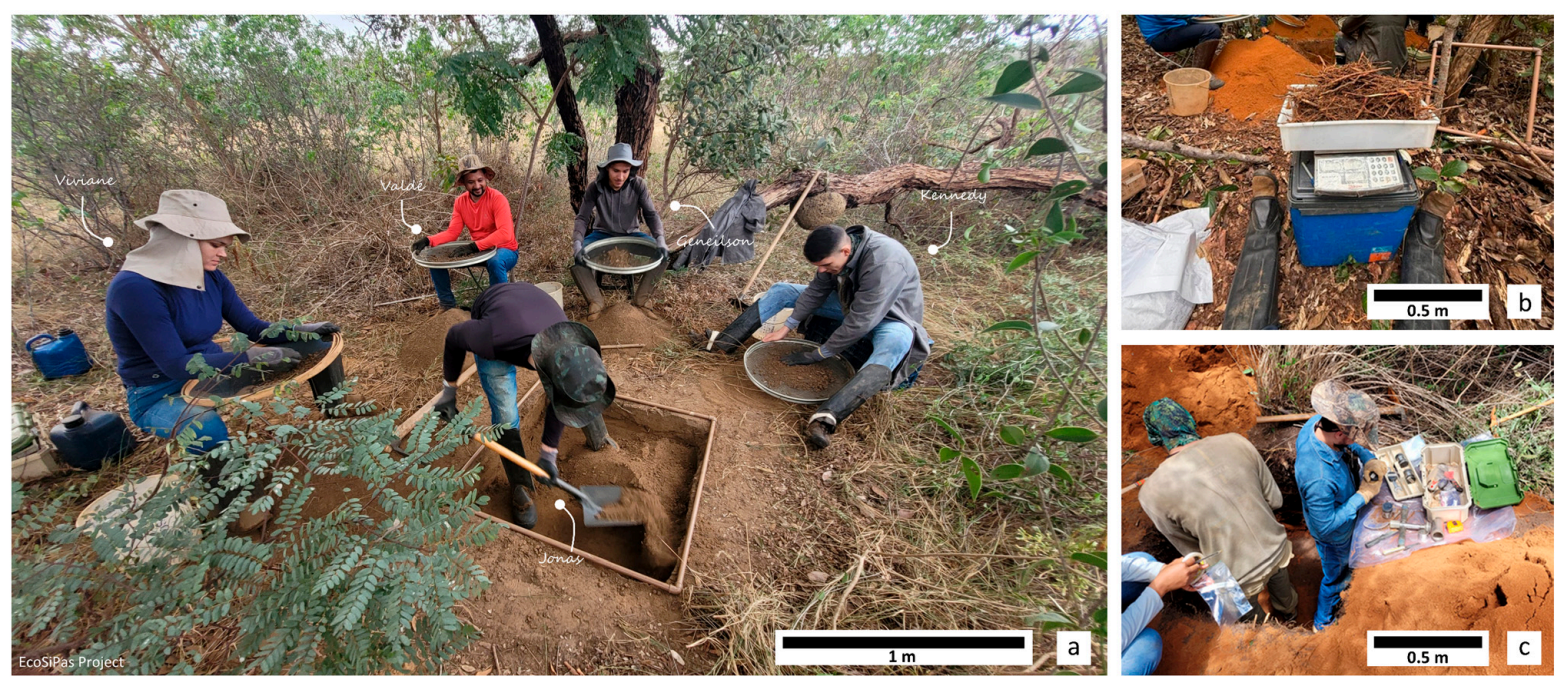
Root sampling (**a**) and weighing (**b**), followed by soil bulk density collection (**c**), in Cerrado *sensu stricto* (CSS) areas, southeastern Brazil.

For root sampling in CSS areas, a trench measuring 1 m × 1 m × 1 m (1 m^3^) was excavated adjacent to the perimeter of each sample plot established for the woody vegetation inventory. Roots were collected in 20 cm depth layers corresponding to 0–20, 20–40, 40–60, 60–80, and 80–100 cm. Using sieves, roots were separated from the soil (Figure 6).

For each layer, the total fresh mass of roots was obtained for each sample. Subsequently, a subsample was taken from each depth layer and oven-dried with air circulation and renewal at 70 ± 2 °C until constant mass was reached (variation ≤ 1%), in order to determine dry mass and thus total dry root biomass per layer [78].

The determination of root dry mass followed the same procedure used for litter dry mass determination.

#### 4.2.3. Soil Physical and Chemical Variables

For fertility and texture analyses, soil samples were collected within the forest inventory sample plots. For bulk density (BD), samples were collected from the trench. Disturbed samples used for fertility analysis were collected using a Dutch auger, forming a composite sample from the 0–20 cm layer. This composite sample was obtained from three subsamples collected along the diagonal of the plot, two near the ends and one at the center (Figure 5). Soil sample preparation used 10-mesh (2 mm) sieves in order to isolate the fine earth fraction, which is the fraction most accessible to plant roots, as described by the Soil Survey Staff [80].

Soil chemical and physical variables included pH, aluminum (Al), organic matter (OM), calcium (Ca), magnesium (Mg), phosphorus (P), potassium (K), sand, silt, and clay contents, as well as bulk density (BD). Chemical and textural analyses were performed in a commercial laboratory that reports using the Manual of Soil Analysis Methods [81].

To determine soil bulk density (BD), undisturbed samples were collected from the 0–20 cm layer directly from the trench profile using cylinders of known volume (Figure 6c). Samples were oven-dried with air circulation and renewal at 103 ± 2 °C until constant mass was reached (variation ≤ 1%), in order to obtain soil dry mass [82]. After drying, the material was sieved to separate the coarse fraction (>2 mm) from the active soil fraction (fine earth < 2 mm), which was used for bulk density determination. The volume of the coarse fraction was determined by water displacement according to Archimedes’ principle. Bulk density was then calculated as the ratio between the dry mass of the fine earth fraction and the effective volume occupied by this fraction (Equation (5)), obtained as the difference between the ring volume and the gravel volume (Equation (6)):

(5) $$BD=\frac{M_{FE}}{V_{FE}}$$

(6) $$V_{FE}=V_{ring}-V_{gravel}$$

where BD = bulk density of the fine earth fraction (g ${cm}^{-3}$); $M_{FE}$ = dry mass of the fine earth fraction (<2 mm) (g); $V_{FE}$ = effective volume of the fine earth fraction (${cm}^{3}$); $V_{ring}$ = total volume of the sampling ring (${cm}^{3}$); and $V_{gravel}$ = volume occupied by the gravel fraction (>2 mm) (cm^3^).

### 4.3. Data Analysis

#### 4.3.1. Floristics, Diversity, and Vegetation Structure

The floristics of CSS were evaluated based on species richness (S) and species diversity. Richness considered the number of families, genera, and species recorded in the vegetation formation.

Vegetation structure was assessed through phytosociological analysis of each vegetation formation and included the variables density, dominance, and frequency of species, and consequently the corresponding Importance Value (IV%). The IV% expresses the level of ecological relevance of each species sampled in each vegetation formation [83] (Table 8) and results from the sum of the relative values of the previously mentioned metrics.

In addition, the ten main species in terms of woody biomass stock were classified according to aluminum tolerance, based on information from the literature [19,26,27,28,29,30].

#### 4.3.2. Biomass Modeling per Unit Area

Models were developed at three hierarchical levels: (a) total biomass (TB), defined as the sum of aboveground woody biomass (AGWB), litter, necromass, and belowground biomass (BGB); (b) total tree biomass (TTB), which includes AGWB and BGB; and (c) woody biomass (AGWB).

Model selection at all levels was initially performed using multiple linear regression (MLR), retaining only the two best models identified through stepwise procedures based on the lowest Akaike Information Criterion (AIC) (Equation (7)) [84], calculated using the mass package [85], and subsequently compared with a simple model based solely on basal area (G).

(7) AIC=−2·log(L)+2·p

where AIC = Akaike Information Criterion; L = maximum value of the likelihood function of the model; and p = number of parameters in the fitted model.

The Variance Inflation Factor (VIF, Equation (8)) was used to assess the presence of multicollinearity among independent variables [34], and was calculated using the car package [86]. VIF provides a robust assessment of collinearity, representing the ratio between the variance of ${\hat{\beta }}_{j}$ in the full model and the variance of ${\hat{\beta }}_{j}$ if fitted in isolation. The minimum possible VIF value is 1, indicating no collinearity. Values greater than 5 or 10 are considered indicative of problematic collinearity [35].

(8) $$VIF({\hat{\beta }}_{j})=\frac{1}{1-R_{X_{j}\;|\;X_{-j}}^{2}}$$

where $VIF({\hat{\beta }}_{j})$ = Variance Inflation Factor; $R_{X_{j}\;|\;X_{-j}}^{2}=$ coefficient of determination from the regression of $X_{j}$ against all other predictor variables. When $R_{X_{j}\;|\;X_{-j}}^{2}$ approaches 1, collinearity is present and VIF assumes high values.

Differences in AIC ($\Delta_{i}$, Equation (9)) were calculated to verify whether alternative models differed from the best model by only one parameter. Models with $\Delta_{i}$ > 10 are considered to have essentially no support [31]. When $\Delta_{i}$ is less than 2, the model with fewer parameters should be selected [32,33].

(9) $$\Delta_{i}=AIC_{i}-AIC_{min}$$

where $\Delta_{i}$ = difference in AIC; $AIC_{i}$ = AIC value of model i; and $AIC_{min}$ = minimum AIC.

The best model presents $\Delta_{i\;}$≡ 0. For the selected models, accuracy was evaluated using the root mean square error (RMSE, Equation (10)) and relative error (RMSE%, Equation (11)) [87], as well as Pearson’s correlation between predicted and observed values (rŷy). Model significance was assessed using the *F* test, and the t statistic was used to test the significance of coefficients. Residual analysis was performed using the Shapiro–Wilk test [88], implemented via the native R function shapiro.test(), to verify the assumption of normality. Residual autocorrelation was evaluated using the Box–Ljung test, implemented via the Box.test() function. Heteroscedasticity was assessed using the Breusch–Pagan test [89], implemented via the bptest() function from the lmtest package [90], as well as by graphical inspection of standardized residuals.

(10) $$RMSE=\sqrt{\frac{1}{n}\sum_{i=1}^{n}{\left({\hat{y}}_{i}-y_{i}\right)}^{2}}$$

(11) $$RMSE\%=\frac{RMSE}{\bar{y}}\cdot 100$$

where RMSE and RMSE% = root mean square error (Mg ha^−1^ and %); n = number of observations; $\hat{y}$ predicted value from the fitted model; y = observed value; and $\bar{y}$ = mean of observed values.

The selected models were validated using k-fold cross-validation implemented in the R package caret [91] and also through an independent manual implementation. In this process, the data were divided into 5 subsets (folds), with one fold at a time set aside for testing, while the model was fitted n = 100 times. For each training subset, the model was fitted and performance metrics including root mean square error (RMSE), mean absolute error (MAE), and coefficient of determination (R^2^) were obtained. The mean values of these metrics were used as estimates of model predictive performance.

All statistical analyses were performed in R software (version 4.5.0) [92], and graphical analyses were conducted using the ggplot2 package [93].

## 5. Conclusions

The inclusion of edaphic variables increased the accuracy of biomass estimates in per-unit-area models in Cerrado *sensu stricto* (CSS) areas in southeastern Brazil, within the Neotropical Savanna domain. This approach reduces taxonomic dependence and the need for wood density databases, which often do not cover the full range of species richness, especially in tropical forests where this variable shows wide variation.

In addition, the use of edaphic variables obtained through relatively simple soil sampling and laboratory analyses makes operations faster and more cost-effective compared to obtaining wood density values, eliminating the need for soil pit excavation and a complete soil characterization. This is particularly relevant when considering belowground biomass. At the same time, these variables allow capturing aspects related to the nutritional adaptations of native vegetation to the characteristic soils of the biome.

In this context, future studies may further improve the understanding of biomass–environment relationships and enhance the predictive performance of biomass models through the integrated evaluation of edaphic attributes associated with soil acidity, nutrient availability, and soil texture.

## Figures and Tables

**Table 1:** Descriptive statistics of biomass components and soil physicochemical attributes in Cerrado *sensu stricto* (CSS) areas (n = 40), northern and northwestern Minas Gerais, Brazil.

Variable	Unit	Average	Minimum	Maximum	Variance	SD	CV%
TB	Mg ha^−1^	45.24	16.30	85.78	300.04	17.32	38.29
TTB	Mg ha^−1^	38.94	12.52	66.65	233.13	15.27	39.21
AGWB	Mg ha^−1^	20.47	3.03	45.56	126.70	11.26	54.98
BGB	Mg ha^−1^	18.47	2.94	57.07	118.32	10.88	58.88
Litter	Mg ha^−1^	5.49	0.40	16.61	17.43	4.18	76.06
Necromass	Mg ha^−1^	0.81	0.00	8.89	2.63	1.62	201.39
G	m^2^ ha^−1^	8.07	1.75	15.62	16.55	4.07	50.39
pH	-	4.21	3.80	4.60	0.03	0.18	4.30
P	mg dm^−3^	1.69	0.80	10.00	2.38	1.54	91.47
Ca	cmolc dm^−3^	0.37	0.20	1.90	0.08	0.28	77.88
Mg	cmolc dm^−3^	0.15	0.10	0.40	0.01	0.07	49.26
K	cmolc dm^−3^	0.10	0.04	0.56	0.01	0.09	90.30
Al	cmolc dm^−3^	0.42	0.10	1.60	0.07	0.27	64.23
OM	%	1.28	0.40	3.50	0.31	0.56	43.97
Sand	%	68.90	32.00	89.00	246.04	15.69	22.77
Clay	%	25.20	10.00	55.00	175.96	13.26	52.64
Silt	%	5.90	1.00	13.00	7.12	2.67	45.22
BD	g cm^−3^	1.33	1.01	1.60	0.02	0.14	10.63
H + Al	cmolc dm^−3^	2.87	1.50	6.40	1.21	1.10	38.28
SB	cmolc dm^−3^	0.61	0.34	2.50	0.14	0.37	61.27
Effective CEC	cmolc dm^−3^	1.03	0.65	2.70	0.16	0.40	39.35
CEC at pH 7	cmolc dm^−3^	3.48	2.15	6.85	1.43	1.19	34.32
m	%	40.83	7.41	73.77	301.46	17.36	42.52
V	%	18.06	6.60	39.05	58.16	7.63	42.23

TB = total biomass; TTB = total tree biomass (AGWB + BGB); AGWB = aboveground woody biomass; BGB = belowground biomass (roots); SD = standard deviation; CV% = coefficient of variation; G = basal area; pH = measured in CaCl_2_; P = phosphorus extracted with Mehlich I; OM = organic matter; Mg = magnesium; K = potassium; Al = aluminum; BD = bulk density; H + Al = potential acidity; SB = sum of bases = Ca + Mg + K; Effective CEC = effective cation exchange capacity = SB + Al; CEC at pH 7 = cation exchange capacity at pH 7 = SB + (H + Al); m = aluminum saturation = $\frac{Al}{Effective\;CEC}\cdot 100$; V = base saturation = $\frac{SB}{CEC\;at\;pH\;7}\cdot 100$.

**Table 2:** Woody biomass and importance value of the ten highest-biomass species in Cerrado *sensu stricto* (CSS), northern and northwestern Minas Gerais, Brazil.

Species	Author	Family	Al-Tolerant	AF	RF	AD	RD	ADo	RDo	IV	AGWB
*Caryocar brasiliense*	Cambess.	Caryocaraceae	Excl. [26]	37.5	2.1	15.3	1.7	0.6526	8.1	4.0	2.8627
*Eugenia dysenterica*	(Mart.) DC.	Myrtaceae	Accum. [27]	72.5	4.1	145.0	15.8	1.1070	13.7	11.2	2.2883
*Qualea parviflora*	Mart.	Vochysiaceae	Accum. [28,29]	65.0	3.7	77.3	8.4	0.6522	8.1	6.7	1.7397
*Terminalia fagifolia*	Mart.	Combretaceae	NA	35.0	2.0	32.0	3.5	0.4339	5.4	3.6	1.5090
*Hymenaea stigonocarpa*	Mart. ex Hayne	Fabaceae	Excl. [26]	47.5	2.7	21.0	2.3	0.2953	3.7	2.9	0.8875
*Qualea grandiflora*	Mart.	Vochysiaceae	Accum. [28,29]	57.5	3.3	40.0	4.3	0.3580	4.4	4.0	0.8339
*Callisthene fasciculata*	Mart.	Vochysiaceae	Accum. [19,30]	12.5	0.7	52.3	5.7	0.3820	4.7	3.7	0.7877
*Vochysia thyrsoidea*	Pohl	Vochysiaceae	Accum. [19,30]	17.5	1.0	11.3	1.2	0.2211	2.7	1.7	0.7556
*Tachigali subvelutina*	(Benth.) Oliveira-Filho	Fabaceae	Excl. [26]	25.0	1.4	12.8	1.4	0.2108	2.6	1.8	0.6141
*Machaerium opacum*	Vogel	Fabaceae	NA	47.5	2.7	18.0	2.0	0.2253	2.8	2.5	0.5942
Total Al-accumulating species			5	225.0	12.8	325.8	35.4	2.7203	33.7	27.3	6.4052
Total Al-excluding species			3	110.0	6.3	49.0	5.3	1.1588	14.4	8.7	4.3643
Total Al-tolerant species (Accum. + Excl.)			8	335.0	19.1	374.8	40.7	3.8791	48.0	36.0	10.7695
Total (top 10 species with highest biomass)			10	417.5	23.8	424.8	46.2	4.5383	56.2	42.1	12.8728
Total (all species)			150	1752.5	100	919.8	100	8.0744	100	100	20.4711

AGWB = aboveground woody biomass (Mg ha^−1^); AF = absolute frequency (%); RF = relative frequency (%); AD = absolute density (individuals ha^−1^); RD = relative density (%); ADo = absolute dominance (m^2^ ha^−1^); RDo = relative dominance (%); IV = importance value (%); Accum. = Al-accumulating; Excl. = Al-excluders; NA = consistent information not available; Haridasan [28]; Haridasan et al. [29]; Haridasan and Araújo [30]; Haridasan [19]; Oliveira et al. [26]; Rodrigues et al. [27].

**Table 3:** Biomass models per unit area fitted for Cerrado *sensu stricto* (CSS) areas in the northern and northwestern regions of Minas Gerais State, Brazil.

Model	Nº
$Y=\beta_{0}+\beta_{1}G+\beta_{2}pH+\beta_{3}Al+\beta_{4}OM+\beta_{5}P+\beta_{6}K+\beta_{7}Ca+\beta_{8}Mg+\beta_{9}Sand+\beta_{10}Silt+\beta_{11}Clay+\beta_{12}BD+\varepsilon$	Full
(a) Modeling total biomass (TB)	
$Y=\beta_{0}+\beta_{1}G+\beta_{2}Mg+\beta_{3}Al+\varepsilon$	M1 *
$Y=\beta_{0}+\beta_{1}G+\beta_{2}Mg+\varepsilon$	M2 *
$Y=\beta_{0}+\beta_{1}G+\varepsilon$	M3 ^#^
(b) Modeling total tree biomass (TTB)	
$Y=\beta_{0}+\beta_{1}G+\beta_{2}Mg+\varepsilon$	M4 *
$Y=\beta_{0}+\beta_{1}G+\varepsilon$	M5 ^#^
(c) Modeling aboveground woody biomass (AGWB)	
$Y=\beta_{0}\cdot G^{\beta_{1}}\cdot e^{\left(\beta_{2}\cdot Mg+\beta_{3}\cdot Sand\right)}+\varepsilon$	M6 *
$Y=\beta_{0}\cdot G^{\beta_{1}}\cdot e^{\left(\beta_{2}\cdot Mg\right)}+\varepsilon$	M7 *
$Y=\beta_{0}\cdot G^{\beta_{1}}+\varepsilon$	M8 ^#^

Y = biomass (Mg ha^−1^); * variables selected by the stepwise method based on the lowest Akaike Information Criterion (AIC); ^#^ simple model. $\beta_{i}$ = parameters; G = basal area (m^2^ ha^−1^); pH (CaCl_2_) = (unitless); Al = aluminum (cmolc dm^−3^); OM = organic matter (%); P (Mehlich I) = phosphorus (mg dm^−3^); K = potassium (cmolc dm^−3^); Ca = calcium (cmolc dm^−3^); Mg = magnesium (cmolc dm^−3^); Sand (%); Silt (%); Clay (%); BD = bulk density (g cm^−3^); ε = error.

**Table 4:** Parameter estimates of biomass models per unit area fitted for Cerrado *sensu stricto* (CSS) in the northern and northwestern regions of Minas Gerais State, Brazil.

Model	Coefficient	SD	X	VIF	*P*_B-P_	*P*_S-W_	*P*_B-L_	$r\hat{y}y$	RMSE	RMSE%	AIC	$\Delta_{i}$
Modeling total biomass (TB)												
M1	$\beta_{0}=24.522$	6.325	*	-	0.94	0.14	0.15	0.76	11.04	24.40	200.11	0
	$\beta_{1}=$ 3.081	0.487	G *	1.13								
	$\beta_{2}=-90.831$	28.152	Mg *	1.16								
	$\beta_{3}=21.727$	7.166	Al *	1.05								
M2	$\beta_{0}=$ 36.526	5.451	*	-	0.76	0.64	0.12	0.69	12.37	27.34	207.21	7.10
	$\beta_{1}=$ 2.986	0.537	G *	1.13								
	$\beta_{2}=-106.160$	30.606	Mg *	1.13								
M3	$\beta_{0}=26.190$	5.185	*	-	0.57	0.07	0.03	0.55	14.24	31.47	216.47	16.36
	$\beta_{1}=2.360$	0.575	G *	-								
Modeling total tree biomass (TTB)												
M4	$\beta_{0}=29.083$	4.643	*	-	0.89	0.12	0.14	0.72	10.54	27.06	194.38	0
	$\beta_{1}=$ 2.781	0.458	G *	1.13								
	$\beta_{2}=-86.823$	26.073	Mg *	1.13								
M5	$\beta_{0}=20.630$	4.374	*	-	0.45	0.09	0.04	0.61	12.01	30.85	202.87	8.49
	$\beta_{1}=2.267$	0.485	G *	-								
Modeling aboveground woody biomass (AGWB)												
M6	$\beta_{0}=1.801$	0.438	*	-	0.57	0.94	0.60	0.94	3.71	18.10	228.30	0
	$\beta_{1}=1.060$	0.079	G *	1.15								
	$\beta_{2}=-1.483$	0.380	Mg *	1.12								
	$\beta_{3}=0.006$	0.002	Sand *	1.04								
M7	$\beta_{0}=2.806$	0.573	*	-	0.10	0.63	0.14	0.93	4.18	20.44	235.99	7.69
	$\beta_{1}=1.047$	0.087	G *	1.11								
	$\beta_{2}=-1.333$	0.413	Mg *	1.11								
M8	$\beta_{0}=2.740$	0.619	*	-	0.01	0.24	0.22	0.90	4.81	23.48	245.09	16.79
	$\beta_{1}=0.966$	0.094	G *	-								

SD = standard deviation of the coefficients; X = independent variables; VIF = Variance Inflation Factor; *P_B-P_* = *p*-value of the Breusch-Pagan test; *P_S-W_* = *p*-value of the Shapiro–Wilk normality test of the residuals; *P_B-L_* = *p*-value of the Box–Ljung test for residual autocorrelation; rŷy = Pearson correlation coefficient between observed and predicted values; RMSE = root mean square error (Mg ha^−1^ and %); AIC = Akaike Information Criterion; $\Delta_{i}$ = differences in AIC; G = basal area (m^2^ ha^−1^); Mg = magnesium (cmolc dm^−3^); Al = aluminum (cmolc dm^−3^); Sand (%). Code: *t*-test significant at the 99% confidence level (*).

**Table 5:** Best-fit equations for estimating biomass in Cerrado *sensu stricto* (CSS) obtained for the northern and northwestern regions of Minas Gerais State, Brazil.

State	Equation	Parameter (Y)
MG	Y=24.522+3.081(G)−90.831(Mg)+21.727(Al)	TB (Mg ha^−1^)
	Y=29.083+2.781(G)−86.823(Mg)	TTB (Mg ha^−1^)
	$Y=1.801\cdot G^{1.060}\cdot e^{\left(-1.483\cdot Mg+0.006\cdot Sand\right)}$	AGWB (Mg ha^−1^)

TB = total biomass; TTB = total tree biomass (AGWB + BGB); AGWB = aboveground woody biomass; BGB = belowground biomass (roots); G = basal area (m^2^ ha^−1^); Mg = magnesium (cmolc dm^−3^); Al = aluminum (cmolc dm^−3^); Sand (%).

**Table 6:** Classification of necromass according to decomposition stages.

Decomposition Class	Characteristic
1—Fresh	Presence of branches and intact wood texture.
2—Initial decomposition	Remnants of bark, without branches and firm wood.
3—Advanced decomposition	Without bark or branches and wood in a moderate to advanced stage of decomposition, crumbling.

Brazilian Forest Service [75].

**Table 7:** Basic density (*ρ*) (g cm^−3^) according to the degree of decomposition and diameter of the woody debris.

Diameter Class (cm)	Decomposition Class		
	1	2	3
2 < d < 4	0.52	0.31	0.28
4 ≤ d < 6	0.63	0.43	0.35
6 ≤ d < 8	0.75	0.45	0.37
8 ≤ d < 10	0.78	0.48	0.45
≥10	0.62	0.44	0.36

Luccas [77].

**Table 8:** Formulas used in the phytosociological analysis of woody vegetation in Cerrado *sensu stricto* (CSS).

Variable	Unit	Formula
Basal area of species *i* (G_i_)	m^2^ ha^−1^	$G_{i}=\sum_{i=1}^{n}\frac{\pi {{\cdot Db}_{i}}^{2}}{40,000}$
Absolute density of species *i* (AD_i_)	individuals ha^−1^	${AD}_{i}=\frac{n_{i}}{A}$
Relative density of species *i* (RD_i_)	%	${RD}_{i}=\frac{{AD}_{i}}{\sum_{i=1}^{n}{AD}_{i}}\cdot 100$
Absolute dominance of species *i* (ADo_i_)	m^2^ ha^−1^	${ADo}_{i}=\frac{G_{i}}{A}$
Relative dominance of species *i* (RDo_i_)	%	${RDo}_{i}=\frac{{ADo}_{i}}{\sum_{i=1}^{n}{ADo}_{i}}\cdot 100$
Absolute frequency of species *i* (AF_i_)	%	${AF}_{i}=\frac{P_{i}}{\sum_{i=1}^{n}P_{i}}\cdot 100$
Relative frequency of species *i* (RF_i_)	%	${RF}_{i}=\frac{{AF}_{i}}{\sum_{i=1}^{n}{AF}_{i}}\cdot 100$
Importance value of species *i* (IV_i_)	%	${IV}_{i}=\frac{{RD}_{i}+{RDo}_{i}+RF}{3}$

Db_i_ = basal diameter of individual *i*, measured at 30 cm above the ground (cm); P_i_ = number of sample plots in which species *i* occurs; n_i_ = number of individuals of species *i* sampled; 40,000: unit conversion factor used to calculate basal area in m^2^ ${ha}^{-1}$ when diameter ($Db_{i}$) is expressed in centimeters.

## Data Availability

The datasets used and/or analysed during the current study are available from the corresponding author on reasonable request.

## Supplementary Materials

The following supporting information can be downloaded at: https://www.mdpi.com/article/10.3390/plants15152261/s1, Table S1: K-fold cross-validation (k = 5, 100 repetitions) of biomass models fitted for Cerrado *sensu stricto* (CSS) in the northern and northwestern regions of Minas Gerais State, Brazil.; Figure S1: K-fold cross-validation of total biomass (TB) models fitted for Cerrado *sensu stricto* (CSS) in the northern and northwestern regions of Minas Gerais State, Brazil (k = 5; 100 repetitions). (a) Comparison between the fitted RMSE and the RMSE obtained by k-fold cross-validation for models M1–M3 (Table 4); (b) Relationship between observed and cross-validated predicted values for models M1, M2, and M3, respectively. Predicted values correspond to the mean out-of-fold predictions obtained across the 100 cross-validation repetitions. The red line represents the fitted linear regression, with the 95% confidence interval.; Figure S2: K-fold cross-validation of total tree biomass (TTB) models fitted for Cerrado *sensu stricto* (CSS) in the northern and northwestern regions of Minas Gerais State, Brazil (k = 5; 100 repetitions). (a) Comparison between the fitted RMSE and the RMSE obtained by k-fold cross-validation for models M4 and M5 (Table 4); (b) Relationship between observed and cross-validated predicted values for models M4 and M5, respectively. Predicted values correspond to the mean out-of-fold predictions obtained across the 100 cross-validation repetitions. The yellow line represents the fitted linear regression, with the 95% confidence interval.; Figure S3: K-fold cross-validation of aboveground woody biomass (AGWB) models fitted for Cerrado *sensu stricto* (CSS) in the northern and northwestern regions of Minas Gerais State, Brazil (k = 5; 100 repetitions). (a) Comparison between the fitted RMSE and the RMSE obtained by k-fold cross-validation for models M6–M8 (Table 4); (b) Relationship between observed and cross-validated predicted values for models M6, M7, and M8, respectively. Predicted values correspond to the mean out-of-fold predictions obtained across the 100 cross-validation repetitions. The blue line represents the fitted linear regression, with the 95% confidence interval.

## Abbreviations

The following abbreviations are used in this manuscript:

CSS	Cerrado *sensu stricto*
TB	Total biomass (AGWB + necromass + litter + BGB)
TTB	Total tree biomass (AGWB + BGB)
AGWB	Aboveground woody biomass
BGB	Belowground biomass
OM	Organic matter
SB	Sum of bases
CEC	Cation exchange capacity
m	Aluminum saturation
IV	Absolute frequency
AF	Importance value
RF	relative frequency
AD	Absolute density
RD	Relative density
ADo	Absolute dominance
RDo	Relative dominance
MLR	Multiple Linear Regression
RMSE	Root mean square error
RMSE%	Relative root mean square error
AIC	Akaike information criterion
VIF	Variance inflation factor
rŷy	Correlation between observed and predicted values
SD	Standard deviation
SEE	Standard error of the estimate
