# Supplementary material for: Total Biomass in the Neotropical Savanna Domain: Stock Estimation and Modeling with Edaphic Variables
Source: Plants (Basel). 2026 Jul 24;15(15):2261. doi: 10.3390/plants15152261 (PMC13468151; doi:10.3390/plants15152261)
Supplement: Supplementary file 1 [file plants-15-02261-s001.zip › plants-4406832-supplementary.pdf]

Table S1. K-fold cross-validation (k = 5, 100 repetitions) of biomass models fitted for Cerrado *sensu stricto* (CSS) in the northern and northwestern regions of Minas Gerais State, Brazil.

Model	RMSE		Generalization gap	MAE	R^2
	Fitted	CV k-fold		CV k-fold	CV k-fold
**Total biomass (TB)**					
M1	11.0386	12.1578	1.1193	9.6721	0.58
M2	12.3678	13.5201	1.1524	10.5208	0.52
M3	14.2372	14.7746	0.5373	12.2575	0.38
**Total tree biomass (TTB)**					
M4	10.5357	11.5417	1.0060	9.5360	0.53
M5	12.0111	12.4904	0.4792	10.2033	0.44
**Aboveground woody biomass (AGWB)**					
M6	3.7055	4.1006	0.3951	3.2633	0.86
M7	4.1831	4.4755	0.2924	3.5186	0.84
M8	4.8054	5.0532	0.2478	4.3021	0.79

RMSE = root mean square error (Mg ha^{-1}); Generalization gap = difference between the RMSE obtained by 5-fold cross-validation (CV k-fold) and the fitted RMSE (Mg ha^{-1}); MAE = mean absolute error (Mg ha^{-1}); R^2 = coefficient of determination.

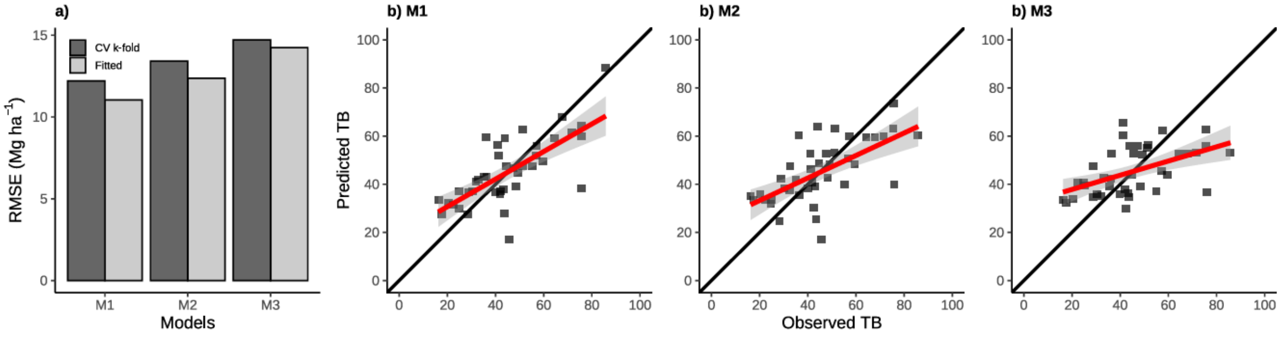

Figure S1. K-fold cross-validation of total biomass (TB) models fitted for Cerrado *sensu stricto* (CSS) in the northern and northwestern regions of Minas Gerais State, Brazil (k = 5; 100 repetitions). **(a)** Comparison between the fitted RMSE and the RMSE obtained by k-fold cross-validation for models M1–M3 (Table 4); **(b)** Relationship between observed and cross-validated predicted values for models M1, M2, and M3, respectively. Predicted values correspond to the mean out-of-fold predictions obtained across the 100 cross-validation repetitions. The red line represents the fitted linear regression, with the 95% confidence interval.

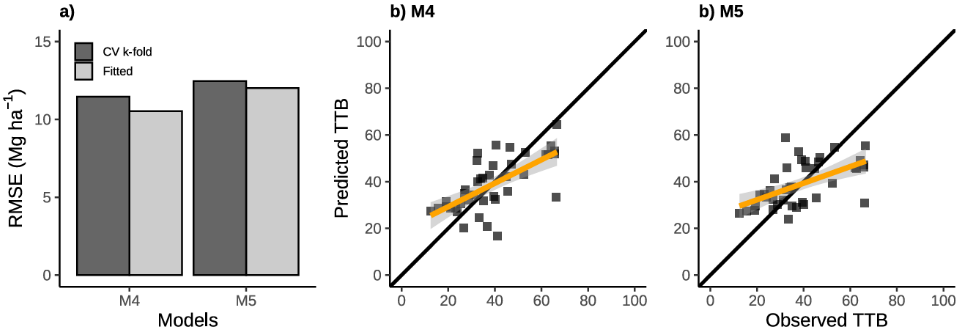

Figure S2. K-fold cross-validation of total tree biomass (TTB) models fitted for Cerrado *sensu stricto* (CSS) in the northern and northwestern regions of Minas Gerais State, Brazil (k = 5; 100 repetitions). **(a)** Comparison between the fitted RMSE and the RMSE obtained by k-fold cross-validation for models M4 and M5 (Table 4); **(b)** Relationship between observed and cross-validated predicted values for models M4 and M5, respectively. Predicted values correspond to the mean out-of-fold predictions obtained across the 100 cross-validation repetitions. The yellow line represents the fitted linear regression, with the 95% confidence interval.

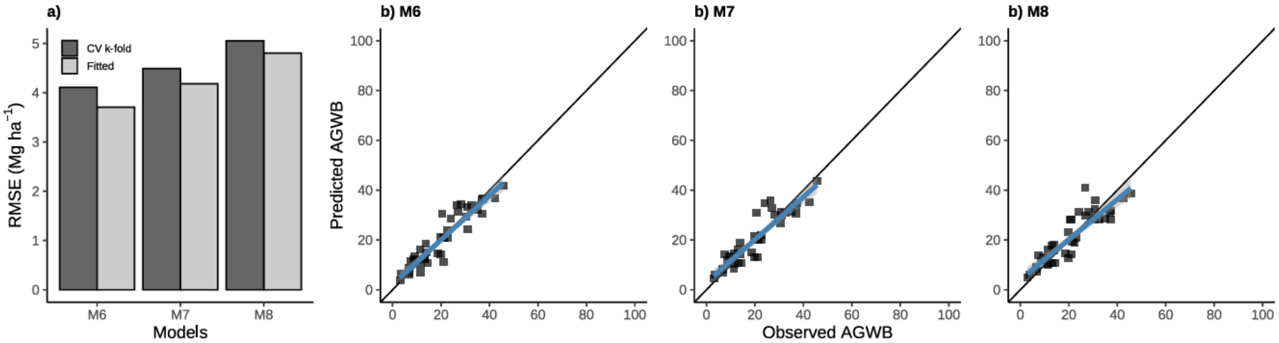

Figure S3. K-fold cross-validation of aboveground woody biomass (AGWB) models fitted for Cerrado *sensu stricto* (CSS) in the northern and northwestern regions of Minas Gerais State, Brazil (k = 5; 100 repetitions). **(a)** Comparison between the fitted RMSE and the RMSE obtained by k-fold cross-validation for models M6–M8 (Table 4); **(b)** Relationship between observed and cross-validated predicted values for models M6, M7, and M8, respectively. Predicted values correspond to the mean out-of-fold predictions obtained across the 100 cross-validation repetitions. The blue line represents the fitted linear regression, with the 95% confidence interval.
